# Altered stability of brain functional architecture after sleep deprivation: A resting-state functional magnetic resonance imaging study

**DOI:** 10.3389/fnins.2022.998541

**Published:** 2022-10-13

**Authors:** Nao-Xin Huang, Zhu-Ling Gao, Jia-Hui Lin, Yan-Juan Lin, Hua-Jun Chen

**Affiliations:** ^1^Department of Radiology, Fujian Medical University Union Hospital, Fuzhou, China; ^2^Department of Nursing, Fujian Medical University Union Hospital, Fuzhou, China; ^3^Department of Cardiovascular Surgery, Fujian Medical University Union Hospital, Fuzhou, China; ^4^School of Medical Imaging, Fujian Medical University, Fuzhou, China

**Keywords:** sleep deprivation, resting-state functional magnetic resonance imaging, dynamic, functional connectivity, functional stability

## Abstract

**Background and aims:**

Resting-state functional magnetic resonance imaging (fMRI) studies using static and dynamic functional connectivity (FC) approaches have revealed brain dysfunction resulting from sleep deprivation (SD). The effects of SD on the stability of brain functional architecture remain unclear. This study investigated the functional stability (FS) changes induced by SD and its association with neurocognitive alterations.

**Materials and methods:**

In this study, we recruited 24 healthy women. All participants underwent two sessions of resting-state fMRI scanning and neurocognitive assessment. The assessments included the Digit Symbol Test, Digit Span Test, Trail-Making Test (TMT), and Complex Figure Test (CFT). Participants completed one session under rested wakefulness (RW) and one session after SD for 24 h. To estimate dynamic FC, we used the sliding window approach; and then, to characterize the FS of each voxel, we measured dynamic FC concordance over time. We used a paired *t*-test to identify differences in FS between RW and SD. To examine the relationship between these changes in FS and alterations in neurocognitive performance, we conducted Spearman’s correlation analyses.

**Results:**

SD affected the performance of the Digit Symbol Test, Digit Span Test, and CFT. Compared with RW, subjects with SD exhibited decreased FS in the bilateral anterior and posterior cingulate gyrus and medial frontal gyrus, right superior frontal gyrus, and cerebellum posterior lobe, while they exhibited increased FS in the bilateral precentral/postcentral gyrus and supplementary motor area, right parahippocampal gyrus and fusiform gyrus, left inferior occipital gyrus, and bilateral cerebellum anterior lobe. After SD, FS changes in the right parahippocampal gyrus and fusiform gyrus were correlated with altered performance in the Digit Symbol Test and CFT.

**Conclusion:**

Our findings showed that the stability of the brain’s functional architecture could be altered by SD. This stability alteration may correspond to multiple neurocognitive domain changes.

## Introduction

Sleep deprivation (SD) is a public health issue in modern societies, which has led to several abnormalities in brain function. For example, SD may impair cognitive functions, including attention, working memory, and learning ability (Krause et al., 2017). SD also is involved in the pathophysiological processes of neurodegenerative disorders, such as Alzheimer’s disease and Parkinson’s disease (Bishir et al., 2020). The neural substrates that SD disturbs brain functions remain to be elucidated.

Several studies have examined the effects of SD on the intrinsic functional organization of brain using resting-state functional magnetic resonance imaging (fMRI). For instance, previous fMRI studies found abnormal functional connectivity density (FCD) in several brain regions in subjects after SD, such as the prefrontal gyrus, precentral gyrus, postcentral gyrus, posterior cingulate gyrus, occipitotemporal cortex, cerebellum anterior lobe, and cerebellum posterior lobe (Kong et al., 2018; Yang et al., 2018). In addition, SD impairs the level of functional communication between distinct brain regions. The results of one study showed that the impact of SD on functional connectivity (FC) was dissociable in the dorsal and ventral default-mode networks (DMN) (Chen W. H. et al., 2018). Another study revealed that SD may decline working memory performance by altering the FC among the DMN, dorsal attention network, and frontoparietal network (FPN) (Dai et al., 2020). Most SD studies using fMRI have adopted the static analytic method, which is based on the assumption that FC remains spatially and temporally stationary throughout the scanning period (Allen et al., 2014). A potential limitation of this static FC approach is that it is too simplistic to capture the dynamic properties of functional communications among the various regions of the brain.

Human brain must dynamically respond to both internal and external stimuli over time. To do so, it uses a highly dynamic system (Hutchison et al., 2013). The dynamic nature of FC is essential for the maintenance of normal brain function, including executive function (Nomi et al., 2017) and attention (Kucyi et al., 2016). Nowadays, dynamic FC approach has been extensively used in neuropsychiatric disorders, such as epilepsy (Liu et al., 2017) and major depressive disorder (Xue et al., 2020). In recent years, existing studies using the dynamic FC have yielded insight into the alterations in brain functional integration caused by SD. For example, one study used the dynamic FC approach and found that subjects after SD spent more time in a functional state with strong subcortical-cortical anticorrelations and remained in a functional state with a globally hypoconnected state for a shorter duration, which has been associated with impaired cognitive performance (Li C. et al., 2020).

Notably, the human brain is a highly complex network composed of billions of neurons, which can achieve a delicate balance between flexibility and stability in changing environments as well as maintain neuronal activity in a stable regime over extended timescales (Slomowitz et al., 2015). Stability is a crucial feature of consciousness, and it maintains a stable and consistent representation of information using distributed neural activity and connectivity patterns over time (Dehaene et al., 2017). Higher temporal stability of the brain network’s modularity is associated with human intelligence (Hilger et al., 2020). The concordance of dynamic FC over time has been measured to characterize the stability of the brain’s functional architecture using resting-state fMRI data (Li L. et al., 2020). The following distribution patterns of the stability of the brain’s functional architecture can be observed: high stability in high-order association regions (such as DMN, FPN, and ventral attentional network) and low stability in unimodal regions (such as somatosensory, motor, visual, and auditory regions). This approach for functional stability (FS) characterization has been used to investigate FS difference between resting and movie-watching states in healthy children and adolescents (Li L. et al., 2020) and applied in several neuropsychiatric field, such as rumination (Chen and Yan, 2021), major psychiatric disorders (Zhu et al., 2020), and amyotrophic lateral sclerosis (Wei et al., 2021).

In this exploratory resting-state fMRI study, we depicted the distribution of FS in individuals during rested wakefulness (RW) status and after SD. We also investigated the association between SD-related FS alteration and neurocognitive change induced by SD.

## Materials and methods

### Subjects

The Ethics Committee of our institution approved this study. We received the written informed consent of all subjects. We recruited 24 women: the average age was 20 years old (±0.81 years old) and the years of education were 13 years (±0.93 years). The number of education years was recorded from primary school. All participants were healthy, demonstrated good sleep habits, and were right-handed. The exclusion criteria were as follows: (1) suffering sleep disorder or any neuropsychiatric diseases; (2) receiving ongoing psychotropic medications treatment; (3) suffering other severe disorders, such as heart failure and malignancies; and (4) presenting contraindications to MRI examination. For at least 2 weeks before participating in this study, subjects were asked to keep a regular sleep-wake schedule. Following the 2 weeks of habitual sleep at home, each subject underwent a neurocognitive assessment and MRI scanning twice: once during the RW status and once after SD. The RW and SD sessions were separated by 2–4 weeks. We counterbalanced the order of RW and SD sessions across all participants to limit the possibility of the session sequence affecting the results (Menz et al., 2012). During the 2- to 4-week intervals, subjects were free to return to their daily routines and work; but subjects were asked to maintain a regular sleep-wake schedule and to refrain from taking any stimulating substances.

During the RW session, subjects needed to stay awake from 8:00 a.m. to 12:00 p.m. on the first day and to sleep from 0:00 a.m. to 8:00 a.m. on the second day. During the SD session, subjects should stay awake for a total of 24 h (8:00 a.m. on the first day to 8:00 a.m. on the second day). In both sessions, the participants stayed in our institution and were allowed to take part in nonstrenuous activities (e.g., reading and watching videos). The intake of stimulating substances and intense physical activity were prohibited. All the neurocognitive assessments and MRI scans were scheduled for 8:00 a.m. to 10:00 a.m. on the second day.

### Neurocognitive assessment

We also performed neurocognitive assessments that covered multiple domains, including the Digit Symbol Test assessing perception, sustained attention, visuomotor coordination, and working memory (van Hoof et al., 1998), Trail-Making Test (TMT) part A assessing visual search and motor speed and TMT part B assessing visual search, executive control, and cognitive flexibility (Bowie and Harvey, 2006), Digit Span Test assessing working memory (Bowden et al., 2013), Complex Figure Test (CFT)-copy trial assessing visuospatial constructional ability, and CFT-immediate and CFT-delay trails assessing visuospatial memory (Shin et al., 2006).

### Acquisition of magnetic resonance imaging data

To obtain resting-state functional images, we followed the multiband slice acquisition method (multiband factor = 4) using a 3.0T scanner (Prisma, Siemens Medical Systems, Erlangen, Germany). To obtain functional data, we applied echo-planar imaging sequences with the following parameters: repetition time = 0.7 s, echo time = 0.03 s, flip angle = 50°, field of view = 228 mm × 228 mm, matrix = 76 × 76, slice thickness = 3 mm (gap = 0), voxel size = 3 mm × 3 mm × 3 mm, axial slice number = 48, and total volume = 600. As instructed, subjects closed their eyes, did not entertain specific thoughts, and held still. To acquire the T1-weighted structural images (resolution = 1 mm × 1 mm × 1 mm), a magnetization prepared rapid gradient echo (MPRAGE) sequence was applied.

### Functional magnetic resonance imaging data preprocessing

We performed fMRI data preprocessing using the Data Processing and Analysis of Brain Imaging (DPABI) toolbox (Yan et al., 2016), which is implemented in the Statistical Parametric Mapping software.^1^ According to previous study (Li L. et al., 2020), we adopted the following preprocessing procedure: (1) discard the first 30 functional volumes; (2) complete the slice timing; and (3) estimate the realignment of the individual’s head motion parameters by calculating the translation in the *x*, *y*, *z* direction and the angular rotation on each axis for each functional volume. No participant showed a head movement greater than the 2 mm translation or 2 degrees of rotation. We also calculated another head motion parameter: frame-wise displacement (FD) reflecting the volume-to-volume changes in head position. (4) We regressed out the nuisance covariates from the fMRI signal, including linear trend, the estimated motion parameters based on the Friston-24 model, white matter signal, and cerebrospinal fluid signal. Additionally, considering the controversy about the resting-state fMRI data preprocessing (Xu et al., 2018; Zhu et al., 2020), we also performed an analysis with the global signal regression (GSR). (5) We spatially normalized functional images. After the individual’s structural image was coregistered with the mean functional image, we adopted the high-level nonlinear warping algorithm—that is, the Diffeomorphic Anatomical Registration Through Exponentiated Lie algebra (DARTEL) method—to segment and normalize the transformed structural image. This calculation yielded the deformation parameters that we then used for the following spatial normalization of the functional image (Ashburner, 2007). (6) We completed band-pass temporal filtering (0.01–0.1 Hz), and (7) smoothed the images with a Gaussian kernel (full width at half maximum = 6 mm).

### Functional stability calculation

We completed the FS calculation using the DPABI toolbox while referring to the previous resting-state fMRI studies (Li L. et al., 2020; Zhu et al., 2020). According to previous study (Li L. et al., 2020), for a given voxel in the brain, its FS was calculated as the concordance of dynamic FC over time of that voxel with the whole brain. In current study, we firstly adopted the following parameters to perform the dynamic FC analysis using the sliding-window method (Li L. et al., 2020; Zhu et al., 2020): the window size was 63 s (=90 TR), the sliding step was 4.2 s (=6 TR), and the window was Hamming type. We also considered the sliding-window parameter-setting controversy (Hutchison et al., 2013). To verify our results, we conducted additional analyses using a different window length (=42 and 84 s), sliding step (=2.1 s), and sliding window type (i.e., the rectangular window). Within each time window, we calculated the Pearson’s correlation coefficients for each gray matter voxel between its time course and the other voxels’ time course; and then, a series of dynamic FC maps across time windows were obtained for that voxel. The above dynamic FC analysis was performed in the voxel-wise manner. After that, using Kendall’s concordance coefficient (KCC) of these dynamic FC maps with time windows as raters, the FS value for each voxel was calculated. The equation for calculating KCC was as follows:


(1) $$\text{KCC}=\frac{\sum_{\text{n}=1}^{\text{N}}{\text{R}}_{\text{n}}^{2}-\frac{1}{\text{N}}\,{\left(\sum_{\text{n}=1}^{\text{N}}{\text{R}}_{\text{n}}\right)}^{2}}{\frac{1}{12}\,{\text{K}}^{2}\,\left({\text{N}}^{3}-N\right)}$$

where K denotes the time window number, N denotes the number of connections of each gray matter voxel with all other gray matter voxels, and Rn denotes the sum of rank for the n-th connection across all time windows. According to FC strength, the connections are ranked across all voxels, in each time window. After the resultant FS maps were derived, they were further standardized into *Z*-scores (Li L. et al., 2020; Zhu et al., 2020). The higher FS value meant that the functional architecture was more consistent and stable over time. Meanwhile, the lower FS value showed an improved ability to achieve frequent and rapid shifts between different brain states.

### Statistical analysis

To investigate the brain’s intrinsic FS profile, we performed a one-sample *t*-test for RW and SD. To identify any FS differences between RW and SD, we conducted a voxel-wise paired *t*-test using the ‘Statistical Analysis Module’ in the DPABI toolbox. The statistical threshold was *P* < 0.05 and was corrected using a Gaussian random field approach (the voxel level was *P* < 0.001). The FD value was included as a covariate in the voxel-wise analysis. After determining the regions with the significant FS difference, we calculated the mean FS values in these regions. We examined the relationship between the FS index and neurocognitive performance according to Spearman’s correlation analyses (the statistical threshold was *P* < 0.05), which was implemented in the SPSS 22.0 software (SPSS, Inc., Chicago, IL, USA).

## Results

The results of the neurocognitive assessment are given in Table 1. Compared with RW, the SD subjects had decreased scores for the Digit Symbol Test (67.5 ± 10.3 vs. 64.5 ± 10.9, *P* = 0.030), the Digit Span Test (14.6 ± 2.6 vs. 13.7 ± 2.8, *P* = 0.021), and the CFT-delay (24.3 ± 7.9 vs. 22.1 ± 6.9, *P* = 0.034). Compared with RW, these subjects also had increased time (seconds) to complete the CFT-delay (67.4 ± 28.2 vs. 84.6 ± 37.2, *P* = 0.022). We did not observe any significant differences in the other neurocognitive assessments.

**TABLE 1:** Neurocognitive assessment results.

	RW	SD	*P-value*
Digit Symbol Test (score)	67.5 ± 10.3	64.5 ± 10.9	**0.030**
Trail-Making Test A (seconds)	20.3 ± 4.7	21.3 ± 5.9	0.396
Trail-Making Test B (seconds)	37.5 ± 11.9	38.0 ± 14.3	0.787
Digit Span Test (score)	14.6 ± 2.6	13.7 ± 2.8	**0.021**
Complex Figure Test-copy (score)	35.2 ± 1.1	34.9 ± 1.8	0.421
Complex Figure Test-copy (time, seconds)	136.6 ± 43.5	133.3 ± 45.1	0.788
Complex Figure Test-immediate (score)	24.5 ± 8.6	22.9 ± 7.3	0.170
Complex Figure Test-immediate (time, seconds)	108.4 ± 41.2	122.5 ± 48.8	0.272
Complex Figure Test-delay (score)	24.3 ± 7.9	22.1 ± 6.9	**0.034**
Complex Figure Test-delay (time, seconds)	67.4 ± 28.2	84.6 ± 37.2	**0.022**

Bold values indicate *P*-value < 0.05.

**FIGURE 1:**
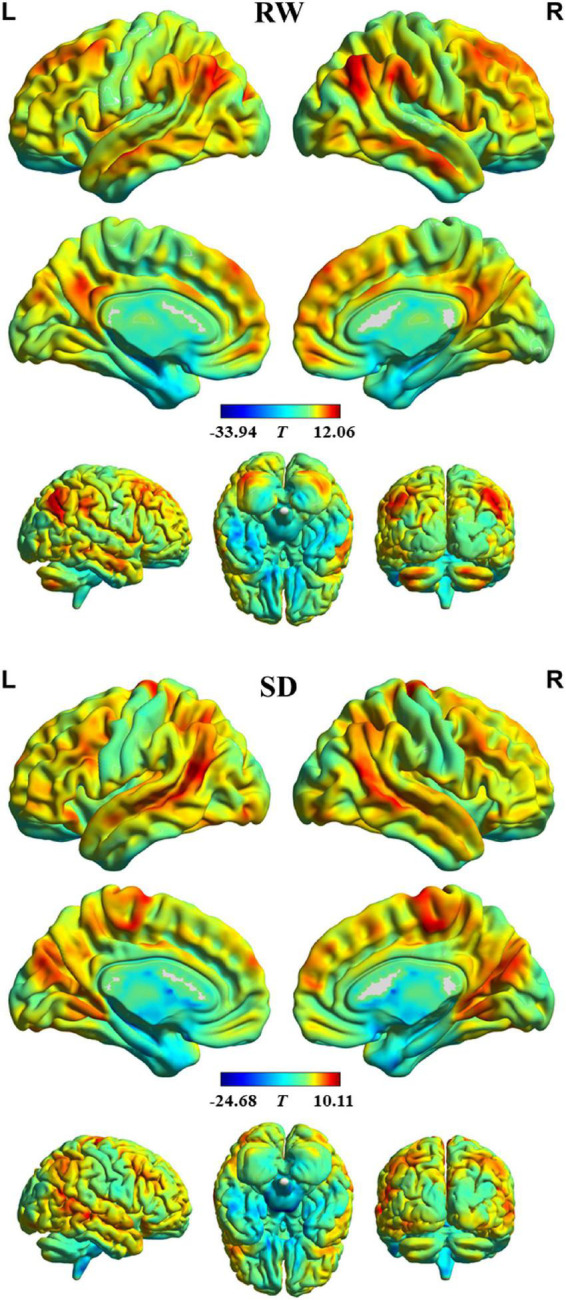
Intrinsic functional stability (FS) profile within rested wakefulness (RW) and sleep deprivation (SD).

The profile of intrinsic FS within each group is shown in Figure 1. In RW, the brain areas with the higher FS value were distributed primarily in the bilateral dorsal lateral prefrontal cortex, medial prefrontal cortex, superior parietal lobule, supramarginal gyrus, angular gyrus, posterior cingulate cortex, lateral temporal cortex, occipitoparietal cortex, anterior insula, and cerebellum posterior lobe. In contrast, brain regions with the lower FS value were found in the bilateral orbitofrontal cortex, precentral and postcentral gyrus, paracentral lobule, parahippocampal gyrus, temporal pole, medial temporal cortex, subcortical nucleus, and cerebellum anterior lobe. In addition, we observed a similar spatial distribution of FS in SD.

**FIGURE 2:**
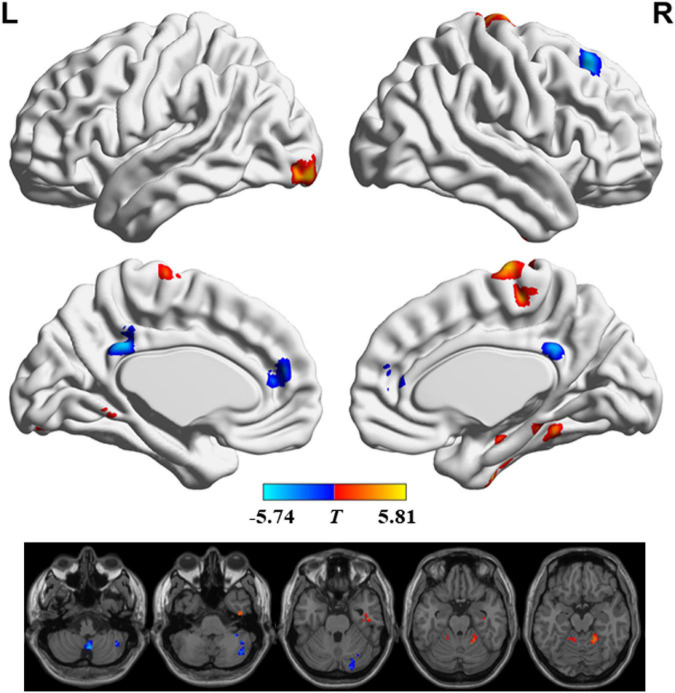
Significant differences in functional stability (FS) between rested wakefulness (RW) and sleep deprivation (SD).

Figure 2 and Table 2 show the regions with significant FS differences. Compared with RW, subjects with SD showed a decreased FS value in the bilateral medial frontal gyrus and anterior cingulate gyrus, bilateral posterior cingulate gyrus, right superior frontal gyrus, and right cerebellum posterior lobe, but an increased FS value in the bilateral precentral/postcentral gyrus and supplementary motor area, right parahippocampal gyrus and fusiform gyrus, left inferior occipital gyrus, right cerebellum anterior lobe and parahippocampal gyrus, and left cerebellum anterior lobe. In addition, we also obtained the similar results when conducting analyses with GSR and different sliding-window parameter settings (see Supplementary Figures 1, 2).

**TABLE 2:** Functional stability (FS) differences between two groups.

Regions	Voxels	Brodmann area	Montreal Neurological Institute (MNI) coordinates			Peak *T*-value
				
			*x*	*y*	*z*	
Bilateral precentral/postcentral gyrus and supplementary motor area	129	6/3/4	9	-18	75	5.68
Right parahippocampal gyrus and fusiform gyrus	42	37/36	45	-15	-27	5.18
Left inferior occipital gyrus	42	18	-30	-95	-12	5.03
Right superior frontal gyrus	65	8	24	30	48	–5.14
Bilateral posterior cingulate gyrus	41	31	-3	-42	33	–4.87
Bilateral medial frontal gyrus and anterior cingulate gyrus	38	10/32/9	-3	45	15	–4.84
Right cerebellum anterior lobe and parahippocampal gyrus	42	36/37	18	-48	-18	5.14
Left cerebellum anterior lobe	36		-9	-48	-15	5.81
Right cerebellum posterior lobe	51		3	-60	-48	–5.74
Right cerebellum posterior lobe	45		27	-90	-39	–5.16
Right cerebellum posterior lobe	82		42	-66	-42	–4.78

Between RW and SD, the changes of mean FS value in right parahippocampal gyrus and fusiform gyrus were negatively correlated with the changes of scores in Digit Symbol Test (*r* = −0.514, *P* = 0.010), and CFT-delay (*r* = −0.483, *P* = 0.017).

## Discussion

This study revealed the altered stability of the brain’s functional architecture after SD. The primary findings were as follows. (1) The neurocognitive assessment revealed that SD affected the multiple domains of neurocognitive function, such as perception, sustained attention, visuomotor coordination, visuospatial memory, and working memory, which was in line with prior studies (Dixit et al., 2012; Gosselin et al., 2017; Batuk et al., 2020). (2) Subjects with SD principally exhibited a decreased FS value in several cognition-related areas (including the medial frontal gyrus, anterior cingulate gyrus, posterior cingulate gyrus, and superior frontal gyrus) and cerebellum posterior lobe; but subjects with SD showed an increased FS value in the sensorimotor-related areas (including the precentral gyrus, postcentral gyrus, and supplementary motor area), visual regions (including the fusiform gyrus and inferior occipital gyrus), parahippocampal gyrus, and cerebellum anterior lobe. (3) After SD, the changes of FS value in the right parahippocampal gyrus and fusiform gyrus were correlated with alterations of performance in Digit Symbol Test and CFT, illustrating that impairment in multiple neurocognitive domains (e.g., sustained attention, working memory, and visuospatial memory) might be associated with altered stability of the brain’s functional architecture.

Following SD, subjects had decreased FS values in several regions of the DMN, such as the posterior cingulate gyrus and the medial frontal gyrus (Chen et al., 2016). Previous SD-related studies also found decreased FCD in the medial frontal gyrus and posterior cingulate gyrus (Yang et al., 2018). The DMN is recognized as a principal component of the brain’s functional architecture and is involved in multiple cognitive functions (Raichle, 2015). The anterior DMN includes the medial frontal gyrus and is responsible for high-level executive functions and decision-related processes (Talati and Hirsch, 2005). The posterior DMN includes the posterior cingulate gyrus and participates in spatiotopographical memory functions (Rolls, 2019). In addition, after SD, subjects showed a decreased FS value in the anterior cingulate gyrus and superior frontal gyrus. Previously, other SD neuroimaging studies have found that the glutamatergic neurotransmission in the anterior cingulate gyrus was altered (Hefti et al., 2013) and that glucose metabolism in the superior frontal gyrus decreased (Wu et al., 2006). These results identified functional abnormalities in these regions, which was consistent with our study. The anterior cingulate gyrus modulates the internal responses, and participates in response selection and cognitively demanding information processing (Devinsky et al., 1995); the superior frontal gyrus is implicated in a range of functional processes, including working memory, introspection, cognitive control, and motor movement (Briggs et al., 2020). Thus, we speculated that a reduction in the FS value in these cognition-related brain areas may be the neural mechanism responsible for SD-induced cognitive dysfunction.

In addition, in keeping with our finding of the reduction in FS value in the cerebellum posterior lobe, functional abnormalities in the cerebellum posterior lobe of the SD subjects have been detected. These abnormalities were reflected by decreased percent amplitude of fluctuation of blood oxygenation–level-dependent signal (Zeng et al., 2020) and a reduced short-range FCD (Kong et al., 2018). By interconnecting with cerebral association and paralimbic cortices, the cerebellum posterior lobe is responsible for functions such as working memory and motor planning (Stoodley and Schmahmann, 2018). A decreased FS value in the cerebellum posterior lobe indicated that its ability to maintain consistent functional coordination with other brain areas may be weakened (Li L. et al., 2020), which may explain impairments in motor planning and working memory that result from SD (Krause et al., 2017; Killgore and Kamimori, 2020).

In contrast, we found that subjects with SD showed an increased FS value in the sensorimotor-related areas, visual regions, parahippocampal gyrus, and cerebellum anterior lobe. Prior research has indicated dysfunction in these regions, such as altered spontaneous brain activity and cerebral activation (Chen L. et al., 2018; Zhao et al., 2019), which aligned with our results. The sensorimotor network (SMN), including the precentral gyrus, postcentral gyrus, and supplementary motor area, is engaged in a premeditated state to ready the brain for motor execution and coordination (Chenji et al., 2016). The fusiform gyrus and inferior occipital gyrus are brain regions included in the visual network (VN), which is involved in visual information processing (Yang et al., 2015). The parahippocampal gyrus is responsible for cognitive processes, including episodic memory and visuospatial processing (Aminoff et al., 2013). The cerebellum anterior lobe forms functional circuits with the sensorimotor areas to support motor execution (Stoodley and Schmahmann, 2018). Previous studies have found that SD weakens brain functions such as motor preparation and execution (Stojanoski et al., 2019), visual perception (Batuk et al., 2020), visuospatial processing (Mathew et al., 2021), and episodic memory (Chai et al., 2020). These dysfunctions may be associated with the increased FS value in the SMN, VN, parahippocampal gyrus, and cerebellum anterior lobe.

Additionally, after SD, the changes of FS value in the right parahippocampal gyrus and fusiform gyrus were correlated with alterations of performance in Digit Symbol Test and CFT. Previous studies found that subjects after SD exhibited poor performance in Digit Symbol Test and CFT (Williamson and Friswell, 2011; Shrimukhi et al., 2020), which agrees with our findings. Correlation analysis suggested that the right parahippocampal gyrus and fusiform gyrus may be the primary cognitive-related brain regions disturbed by SD.

This study had several limitations. First, this study had a relatively small sample size, which reduced its statistical power. Second, this study only included female participants. It has been suggested that the effect of SD on brain function differs between genders (Dai et al., 2012). To avoid the impact of gender (as a confounding factor) on our results, we only included female participants in current study. However, both female and male subjects could be included in future study, to identify gender-related difference in FS alteration following SD. Third, the FS change was evaluated only after SD in this study and represented the consequence of SD. As previously reported (Zhu et al., 2019), however, multiple functional measurements across distinct time points during the SD period could contribute to a better understanding of how SD dynamically affects brain functions.

This study elucidated the effects of SD on brain functional architecture from a stability perspective. Subjects with SD exhibited decreased stability in the DMN, anterior cingulate gyrus, superior frontal gyrus, and cerebellum posterior lobe, but they showed increased stability in the SMN, VN, parahippocampal gyrus, and cerebellum anterior lobe. The altered stability of brain functional architecture may be responsible for the neural substrates about SD-induced dysfunctions in multiple cognitive domains.

## Data availability statement

The original contributions presented in this study are included in the article/Supplementary material, further inquiries can be directed to the corresponding authors.

## Ethics statement

The studies involving human participants were reviewed and approved by the Ethics Committee of Fujian Medical University Union Hospital. The patients/participants provided their written informed consent to participate in this study.

## Author contributions

N-XH and Z-LG: data curation, formal analysis, investigation, and writing—original draft. J-HL: data curation and investigation. Y-JL: conceptualization, supervision, and writing—review and editing. H-JC: conceptualization, data curation, formal analysis, funding acquisition, investigation, project administration, supervision, visualization, and writing—review and editing. All authors contributed to the article and approved the submitted version.

## Footnotes

1
http://www.fil.ion.ucl.ac.uk/spm

## Supplementary material

Supplementary Figure 1 Significant functional stability differences when performing analysis with global signal regression (GSR).

Supplementary Figure 2 Significant functional stability differences when performing analyses with different sliding-window parameter settings. **(A)** Window size = 42 s, sliding step = 4.2 s, and Hamming window type; **(B)** window size = 63 s, sliding step = 2.1 s, and Hamming window type; **(C)** window size = 84 s, sliding step = 4.2 s, and Hamming window type; **(D)** window size = 63 s, sliding step = 4.2 s, and rectangular window type.

## Abbreviations

CFT: Complex Figure Test
DMN: default-mode network
FC: functional connectivity
FCD: functional connectivity density
fMRI: functional magnetic resonance imaging
FPN: frontoparietal network
FS: functional stability
RW: rested wakefulness
SD: sleep deprivation
SMN: sensorimotor network
TMT: Trail-Making Test
VN: visual network.
