# Supplementary figures and images for: Altered stability of brain functional architecture after sleep deprivation: A resting-state functional magnetic resonance imaging study
Source: Front Neurosci. 2022 Oct 13;16:998541. doi: 10.3389/fnins.2022.998541 (PMC9606579; doi:10.3389/fnins.2022.998541)

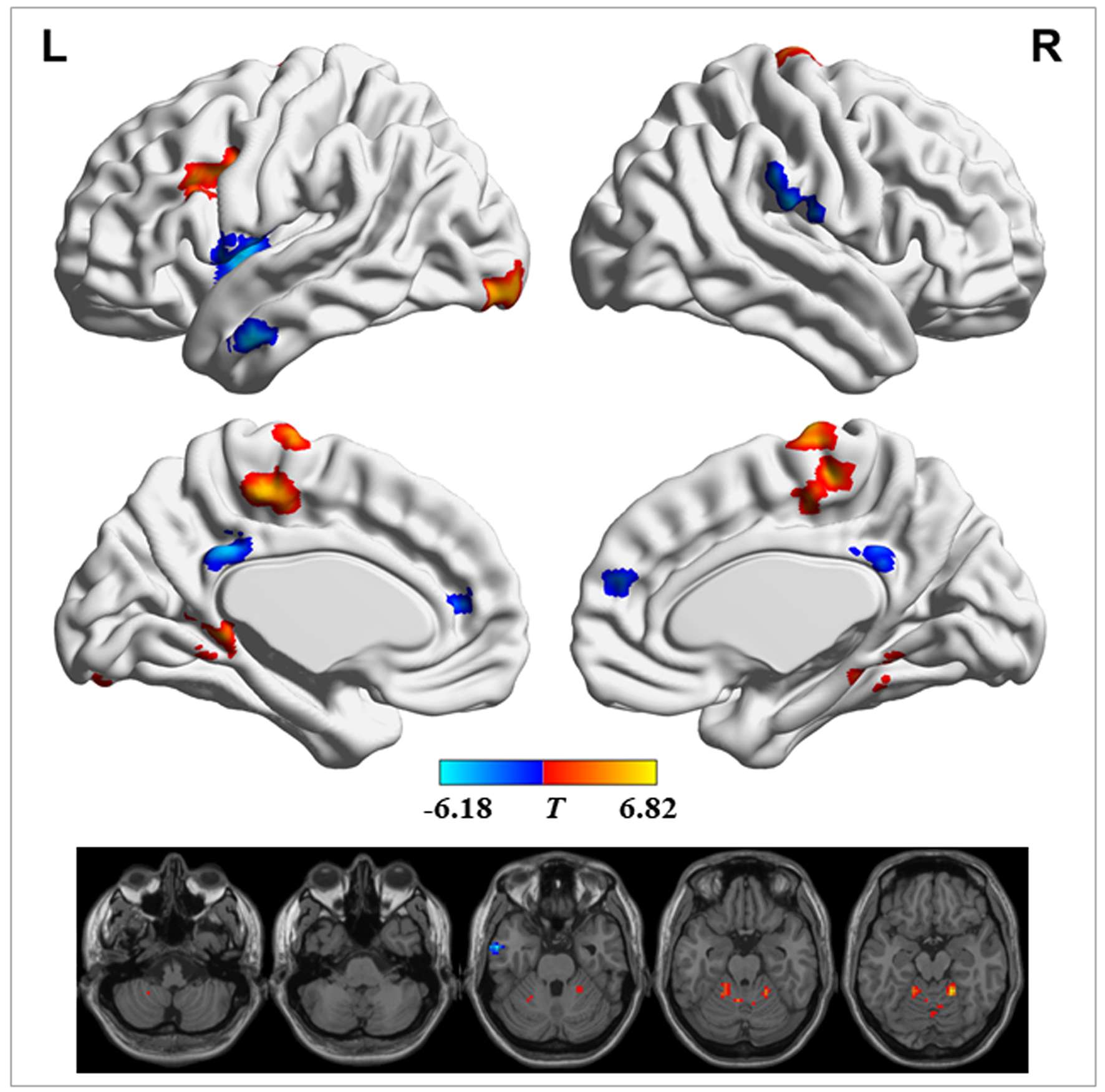

Supplement: Supplementary Figure 1 — Significant functional stability differences when performing analysis with global signal regression (GSR). [file Image_1.TIF]

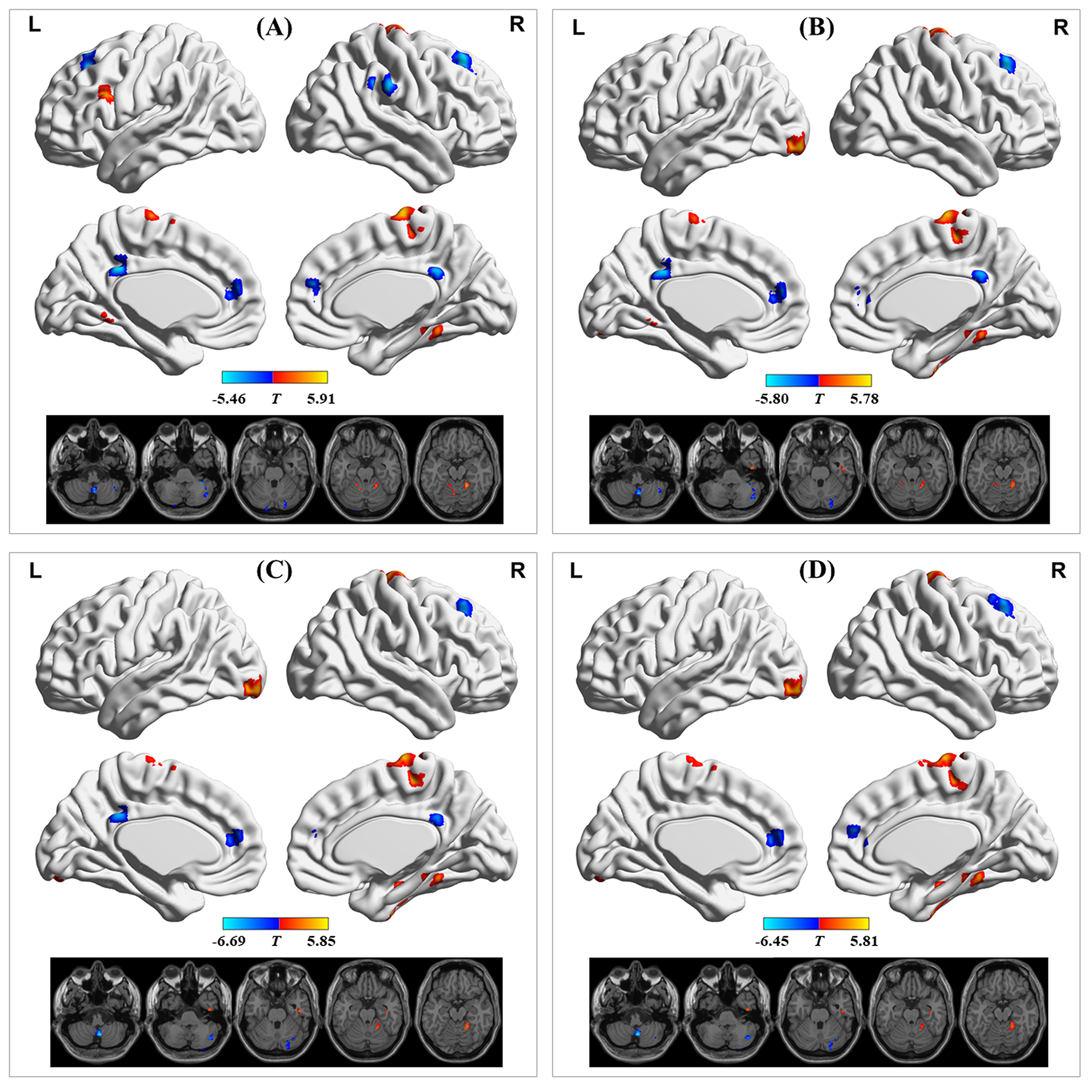

Supplement: Supplementary Figure 2 — Significant functional stability differences when performing analyses with different sliding-window parameter settings. (A) Window size = 42 s, sliding step = 4.2 s, and Hamming window type; (B) window size = 63 s, sliding step = 2.1 s, and Hamming window type; (C) window size = 84 s, sliding step = 4.2 s, and Hamming window type; (D) window size = 63 s, sliding step = 4.2 s, and rectangular window type. [file Image_2.TIF]
